# Bilateral lung herniation with parenchymal infarction following clamshell thoracotomy for lobar lung transplantation: a case report

**DOI:** 10.1186/s13019-025-03361-6

**Published:** 2025-02-18

**Authors:** Janis Tavandžis, René Novysedlák, Jiří Pozniak, Monika Švorcová, František Mošna, Jaromír Vajter, Zuzana Ozaniak Střížová, Vojtěch Suchánek, Jan Šimonek, Jiří Vachtenheim, Robert Lischke

**Affiliations:** 1 Prague Lung Transplant Program, 3rd Department of Surgery, First Faculty of Medicine, Charles University and Motol University Hospital, Prague, Czech Republic; 2 Department of Anaesthesiology, Resuscitation and Intensive Care Medicine, Second Faculty of Medicine, Charles University and Motol University Hospital, Prague, Czech Republic; 3 Department of Immunology, Second Faculty of Medicine, Charles University and Motol University Hospital, Prague, Czech Republic; 4 Department of Imaging Methods, Second Faculty of Medicine, Charles University and Motol University Hospital, Prague, Czech Republic

**Keywords:** Clamshell thoracotomy, Lung hernia, Lung transplantation

## Abstract

**Background:**

Pulmonary hernia is a rare condition characterized by the protrusion of lung tissue through a chest wall defect. Trauma and thoracic surgery are the most common causes of acquired lung hernias. We present an unusual case of (sequential) bilateral lung herniation with parenchymal infarction after bilateral lobar lung transplantation.

**Case presentation:**

A 50-year-old female, wait-listed as high-urgency candidate, with a body mass index (BMI) of 29 kg/m^2^ underwent a bilateral lobar lung transplantation for pulmonary fibrosis through a clamshell thoracotomy approach. Due to a size mismatch, stapler resection of the segment 3 and the middle lobe of the right lung, as well as an upper left lobectomy was required. The chest was closed with 3 braided non-absorbable pericostal sutures on each side. Sternal osteosynthesis was performed with a titanium sternal splint along with 7 self-tapping screws with a length of 18 mm. On the posttransplant day (PTD) 18, patient’s clinical condition deteriorated. Physical examination didn’t reveal any palpable subcutaneous chest resistance. However, a computed tomography (CT) scan showed a herniation of the segment 6 of the right lung. During acute surgical revision, perioperative finding revealed posterior pericostal suture failure. Therefore, a stapler resection was performed due to the infarction of the herniated segment. On the PTD 36, herniation of the left lung parenchyma was detected by acute CT scan. The protruding vital parenchyma was surgically repositioned without necessity of resection. Two posterior pericostal sutures were broken, and distal part of sternal splint detached. Thoracotomy was closed using 5 braided non-absorbable sutures. Sternum was re-osteosynthesized with the STRATOS™ system. After 3 months of intensive postoperative care, the patient was transferred to the rehabilitation department. She was discharged on the PTD 99. After 20 months of follow-up, lung function remains stable without the need for oxygen support.

**Conclusion:**

Clamshell incision remains ultimate approach in thoracic surgery. However, pulmonary herniation after clamshell thoracotomy is a rare complication and may manifest as acute respiratory distress syndrome with an inflammatory response. In these cases, CT scan should be always considered, even if no palpable pathology of chest is present.

## Background

Pulmonary hernia is a rare condition wherein lung tissue protrudes through a defect in the chest wall. It can be classified as either congenital or acquired. Acquired hernias manifest spontaneously, following trauma, or due to chest wall pathology. The risk factors for acquired hernias are chest trauma and prior surgical interventions [1]. Predisposing factors may include malignancy, chronic obstructive pulmonary disease, obesity, diabetes mellitus, and steroid use [2–4]. 

The clinical presentation can be completely asymptomatic in small hernias. Usually, there is a palpable subcutaneous mass that enlarges during the Valsalva maneuver. Large hernias pose a risk of incarceration and strangulation, requiring prompt surgical intervention and potential resection of the involved damaged lung parenchyma [5]. The preferred diagnostic method is computed tomography (CT) due to its high specificity and sensitivity [6]. 

The management of pulmonary hernias is widely influenced by their size. Small, asymptomatic hernias may be managed conservatively without intervention, whereas large and symptomatic hernias require surgical management. Chest wall surgery is a potential predisposing factor for pulmonary hernia development. Therefore, materials such as Gore-Tex or polypropylene meshes, rib splints, and biological implants are commonly used for the reconstruction of defects after chest wall resection [7–11]. 

## Case presentation

A 50-year-old female (body mass index, BMI 29 kg/m^2^) with pulmonary fibrosis, rheumatoid arthritis, asthma, arterial hypertension, and diabetes mellitus had been hospitalized for almost 44 days at department of pneumology due to respiratory infection with the need of high flow nasal oxygen (HFNO). Given the disease progression and decline in lung function, she was evaluated by multidisciplinary team and subsequently listed as the urgent candidate for lung transplantation.

The patient underwent bilateral lobar lung transplantation in January 2023, following 24 days on the waiting list, with perioperative central veno-arterial extracorporeal membrane oxygenation support. Clamshell thoracotomy approach was performed at the fourth intercostal space. Stapler resection of the right S3 segment and right middle lobe as well as upper lobectomy of the left lung had been performed due to size mismatch on the back-table prior to implantation. The closure of the chest involved the application of three pericostal non-absorbable polyester sutures and absorbable polyglactine 910 muscle sutures on each side to seal the intercostal spaces. Sternum fixation was achieved using the Synthes^®^ Titanium Sternal Fixation System plate (DePuy Synthes, Zuchwil, Switzerland) secured with seven self-tapping screws (18 mm). Subcutaneous sutures were completed in two layers, utilizing (Ethicon VICRYL™) absorbable suture, and skin closure was performed with a stapler.

Postoperatively, the patient was transferred to intensive care unit (ICU) requiring mechanical ventilation for a total of 33 days. Postoperative course was complicated by renal insufficiency (without the need for hemodialysis), septic episodes, ICU myopathy, and repeated bronchoscopy for airway clearance, followed by tracheostomy performed through an open surgical approach. Due to a constant nausea and vomiting, percutaneous endoscopic gastrostomy/jejunostomy was utilized to ensure the nutritional support and prevention of aspiration. Other complications that arose during the post-transplantation period included infections and colonization with multidrug-resistant bacteria, such as *Pseudomonas aeruginosa, Enterobacter cloacae* producing extended-spectrum beta-lactamase (ESBL), and *Klebsiella pneumoniae* ESBL, as well as cholecystitis and heart failure with reduced ejection fraction.

**Fig. 1:**
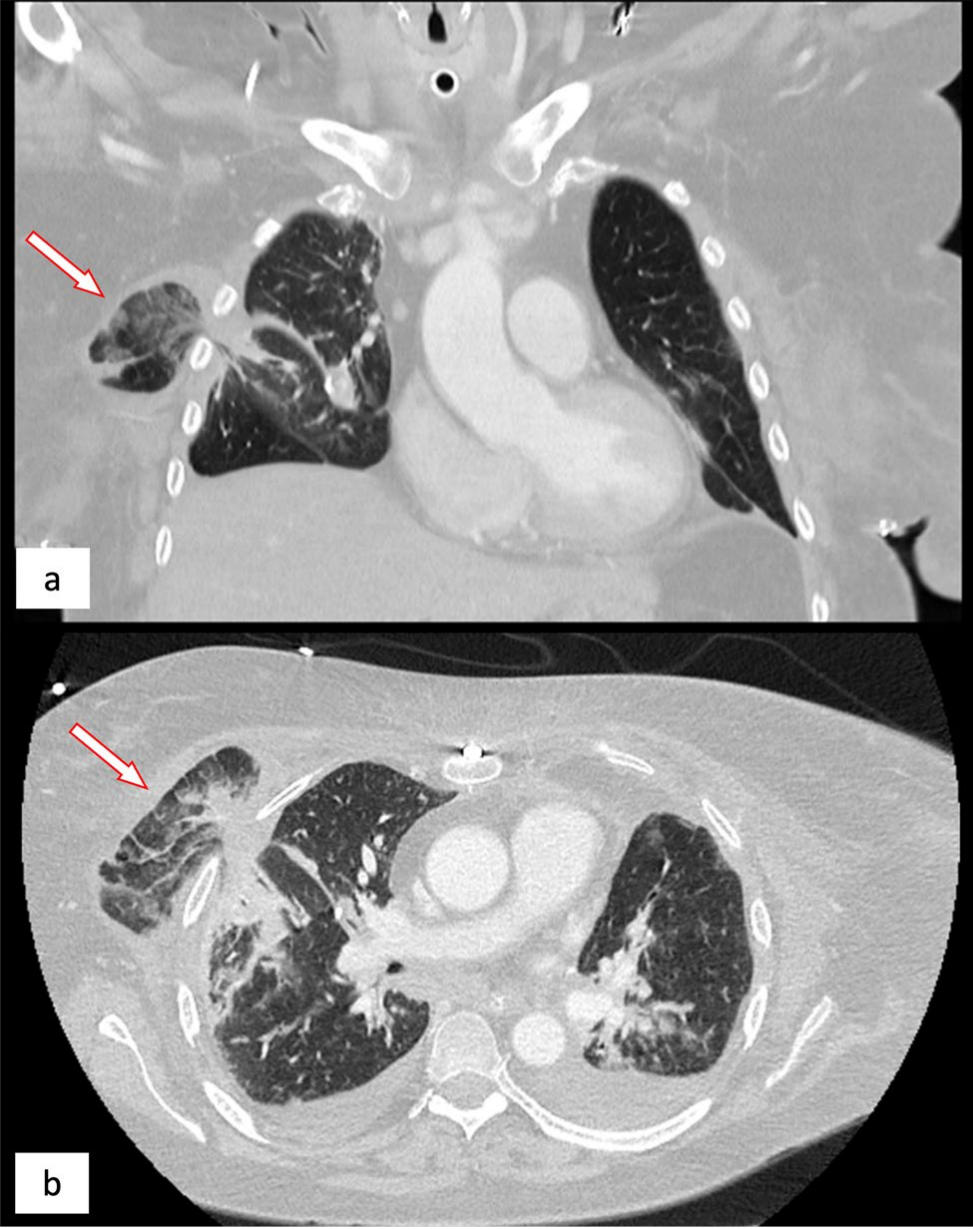
Chest CT scan, coronary (**a**) and transversal (**b**) axis. Arrows highlighting right lung herniation at 4th intercostal space

**Fig. 2:**
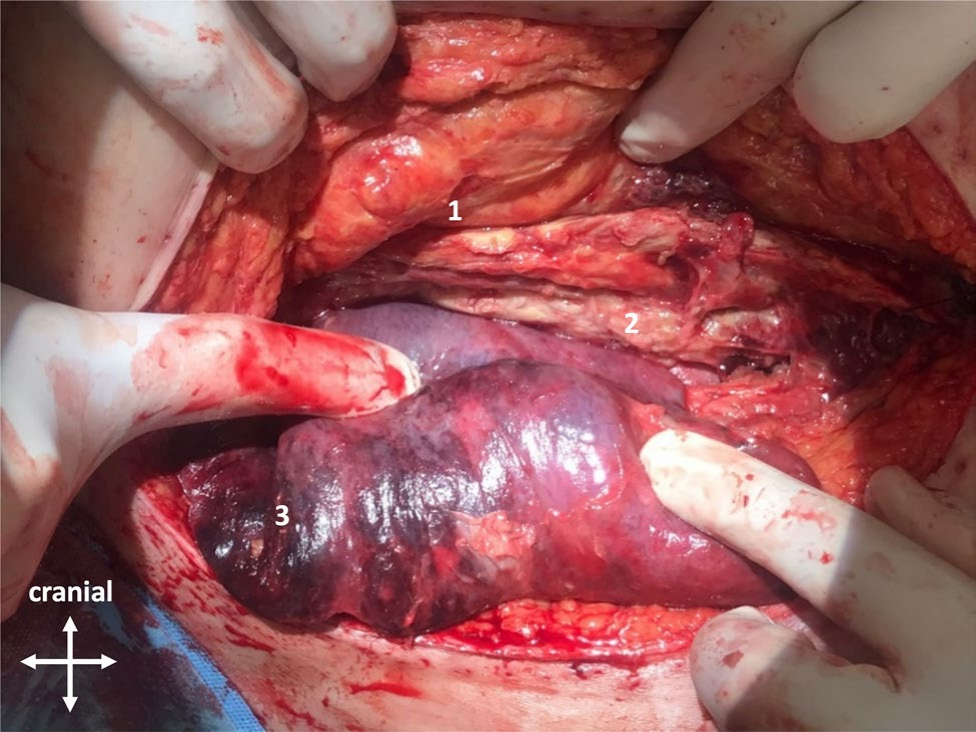
Right lung herniation through the chest wall defect at 4th intercostal space. Parenchyma is livid, congested, and infarcted. Craniocaudal view. 1– subcutaneous tissue, 2–4th rib, 3– infarcted lung parenchyma

On the posttransplant day (PTD) 18, while the patient was still sedated and mechanically ventilated, the worsening clinical condition and elevated inflammatory parameters prompted a chest CT, revealing herniation of segment 6 of the right lung (Fig. 1). An urgent revision was performed with a finding of a failure of the posterior pericostal suture. The pulmonary parenchyma was infarcted and venous congestion was already present (Fig. 2). Therefore, stapler resection of the entire protruding tissue (90 × 30 mm) was necessary.

**Fig. 3:**
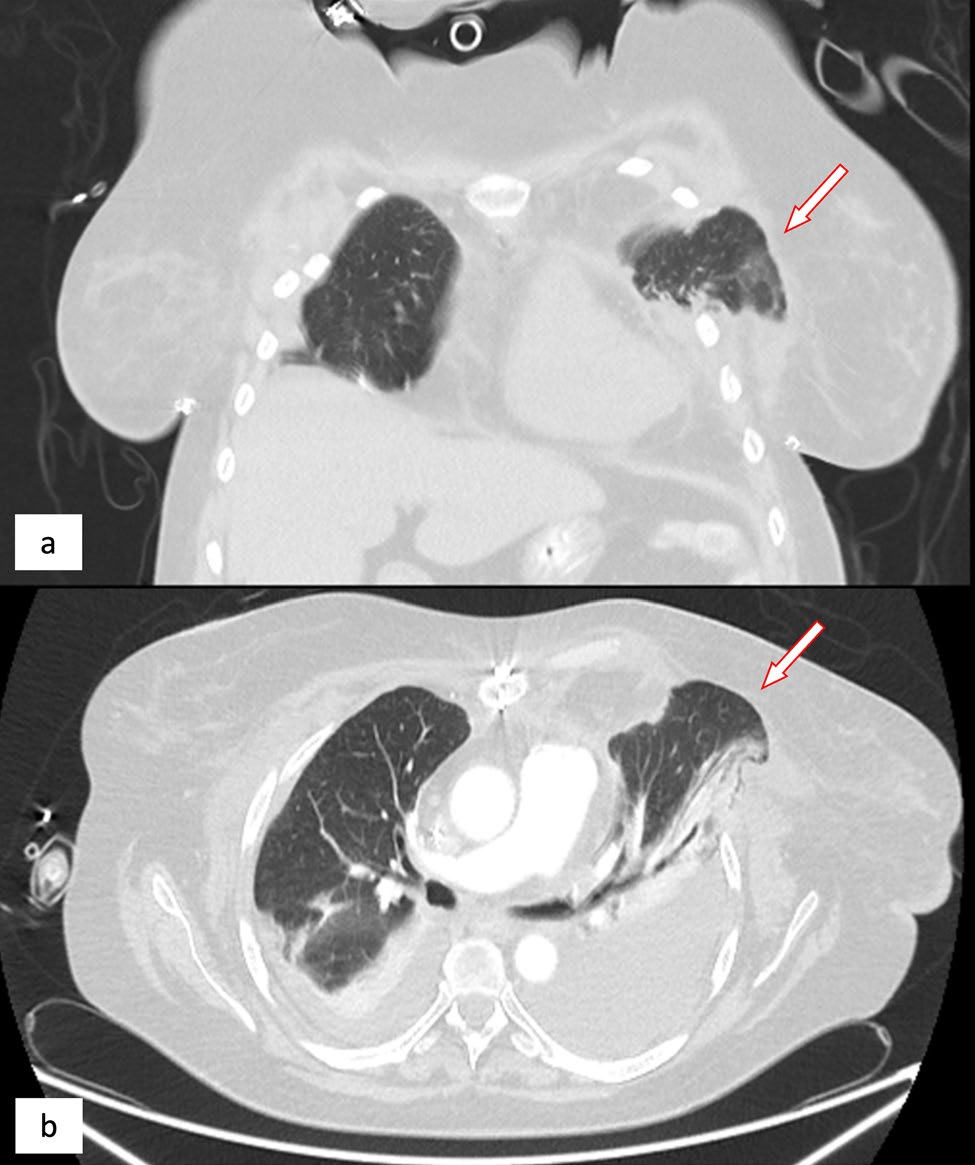
Chest CT scan, coronary (**a**) and transversal (**b**) axis. Arrows highlighting left lung herniation at 4th intercostal space

**Fig. 4:**
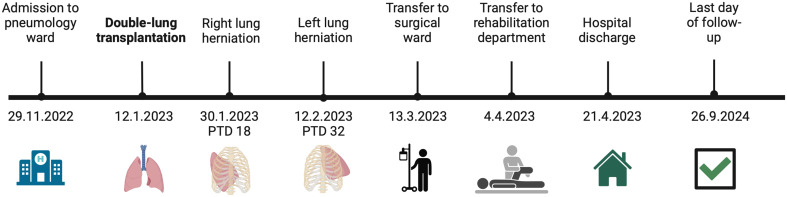
Timeline illustrating the clinical course. Created in BioRender.com

On the PTD 32, inflammatory parameters increased again, with the development of septicaemia. A chest CT (Figs. 3 and 4), which was done two days after the outburst of symptoms, showed herniation of the left lung. Urgent surgical revision revealed two completely loosened posterior pericostal sutures and a completely detached distal end of the Synthes^®^ Titanium Sternal Fixation System plate with the missing anterior sternal cortex. The protruding vital lung parenchyma was repositioned without the need of resection and thoracotomy closed using 5 pericostal sutures. Sternal re-osteosynthesis was performed with a triple STRATOS™ (MedXpert GmbH, Eschbach, Deutschland) sternal plate.

Weaning was successfully managed through HFNO, and rehabilitation initiated as inflammatory parameters decreased. On PTD 33, patient was transferred to a surgical ward. Subsequent course was uneventful, and nearly 3 months post-transplantation, the patient was transferred to a rehabilitation department. Patient was discharged on PTD 99 and as of the follow-up date on September 26th, 2024 (PTD 623), the patient remains in a stable condition with chronic lung allograft dysfunction grade 0p, does not require oxygen therapy and consistently attends follow-up appointments.

## Discussion and conclusions

Pulmonary parenchymal herniation is a rare condition which occurrence is affected by several risk and predisposing factors, including thoracic surgery, obesity, and diabetes mellitus. In our case, it developed after bilateral lobar lung transplantation through clamshell approach. The diagnostic method of choice is chest CT for its ability to determine the degree of parenchymal herniation. Although a CT scan was promptly performed after the onset of respiratory distress and an inflammatory response prior to the first revision for right-sided pulmonary herniation, it is notable that the second herniation took nearly two days to reach a proper diagnosis. No parenchymal infarction after that time was present. At first, such complication as bilateral herniation might be mistaken for incipient pulmonary infection or acute rejection and early diagnostic process plays crucial role. Infarction of pulmonary parenchyma can potentially result in lung cavitation, abscess formation, or bacterial pneumonia. Risk factors include infarctions larger than 4 cm, elderly patients, chronic lung disease, and heart failure [12]. 

In our case, the ultimate cause of pulmonary hernia was insufficient intercostal suturing, that was accompanied by many risk factors, such as patient’s BMI 29 kg/m^2^, medical history of steroid diabetes mellitus due to chronic corticosteroid medication for rheumatoid arthritis, prolonged deconditioning during hospitalization for a lung infection before transplantation, postoperative mechanical lung ventilation with increased intrathoracic pressure, and impaired wound healing due to immunosuppression and corticoid therapy [2, 13]. The early postoperative respiratory and mobility rehabilitation is important and necessary [14]. There is no evidence concerning the impact of early rehabilitation on the risk of lung herniation.

The anterior chest wall is anatomically weakened site, with intercostal spaces farther apart, making it a herniation-prone area [3]. Intercostal muscles, which strengthen and fill the intercostal spaces are disrupted during lung transplantation with clamshell thoracotomy, along with the sternum. Prolonged immobility and inactivity lead to decrease of muscle mass and strength loss. In our case, all of these predisposing factors combined with extensive surgical procedure led to postoperative complications, classifying as grade III in Clavien-Dindo system [15]. 

Proper wound closure, adequate approximation of intercostal spaces, and their fixation are crucial. This is even more important in high-risk patients with obesity and diabetes. There are several techniques for chest wall defect closure, including use of plates, spinal fixation and meshes. Therapy of choice should be individualized based on the case-by-case basis. Pulmonary hernia is a rare complication after thoracic surgery. Large symptomatic hernias require surgical revision with chest wall defect repair using artificial materials. The risk of any hernia is its incarceration, which can be complicated by parenchymal infarction, subsequent infection, and abscess formation. In patients after extensive thoracic procedures with the development of sudden respiratory distress, a CT scan should be performed to exclude pulmonary hernia, even in the absence of a newly palpable mass in the wound area or significant findings on chest X-ray.

## Data Availability

No datasets were generated or analysed during the current study.

## Abbreviations

BMI: body mass index
HFNO: high flow nasal oxygen
ICU: intensive care unit
PTD: post-transplant day
CT: computed tomography
ESBL: extended spectrum beta lactamase
